# A Deserved Tribute

**Published:** 1918-12

**Authors:** 


### A Deserved Tribute.

A subscription list has been started at McKinney, Texas, for the erection of a sanitarium as a memorial to the Collin County boys who gave their lives for their country in the World War. One citizen headed the list with a $1000 subscription, and it is believed that the building erected will be a most fitting memorial to the soldier boys. This plan, or something like it, should be adopted in many counties in the State that are now without sufficient hospital facilities.

---

Have you received a bill for your subscription to the Texas Medical Journal within the last month? If so, what did you do with it? Perhaps you gave it the same attention some of your least appreciated patrons give to your bills. Treat others as you would have them treat you. If you have not paid your subscription send it in at once, so that it will reach the editor before Christmas.

